# Colonic transendoscopic tube-delivered enteral therapy (with video): a prospective study

**DOI:** 10.1186/s12876-020-01285-0

**Published:** 2020-05-06

**Authors:** Ting Zhang, Chuyan Long, Bota Cui, Heena Buch, Quan Wen, Qianqian Li, Xiao Ding, Guozhong Ji, Faming Zhang

**Affiliations:** 1 Medical Center for Digestive Diseases, the Second Affiliated Hospital of Nanjing Medical University, Nanjing, 210011 China; 2 Key Lab of Holistic Integrative Enterology, Nanjing Medical University, Nanjing, 210011 China; 3 Department of Cardiovascular Medicine, The Second Affiliated Hospital of Nanchang University, Nanchang, 330006 China

**Keywords:** Transendoscopic enteral tubing, Fecal microbiota transplantation, Enema, Colonoscopy, Method

## Abstract

**Background:**

Colonic transendoscopic enteral tubing (TET) refers to colonic transendoscopic tube-delivered enteral therapy. Colonic TET has been successfully used for frequent colonic administration of drugs or multiple fecal microbiota transplantations (FMTs). This prospective observational study aimed to evaluate possible factors affecting methodology, feasibility and safety of colonic TET.

**Methods:**

Patients who underwent colonic TET at our center from October 2014 to November 2018 were included. The feasibility, efficacy, and safety of TET were evaluated.

**Results:**

In total, 224 patients were analyzed. The success rate of TET was 100%. The median retention time of TET tube within the colonic lumen was 8.5 (IQR 7–11) days in 158 patients with tube falling out spontaneously, and the maximum retention time was up to 28 days. These patients were divided into the short-retention group (≤ 8.5 days) and the long-retention group (> 8.5 days). Univariate and multivariate analysis demonstrated that the type of endoscopic clip (*p* = 0.001) was an independent factor for the retention time. The larger clips as well as a greater number of clips significantly affected the retention time (*p* = 0.013). No severe adverse event was observed during and after TET.

**Conclusions:**

Colonic TET is a feasible, practical, and safe colon-targeted drug delivery technique with a high degree of patients’ satisfaction. Two to four large endoscopic clips are recommended to maintain stability of the TET tube within the colon for over 7 days.

## Background

Fecal microbiota transplantation (FMT) has shown the therapeutic potential in many microbiota-related diseases beyond *Clostridium difficile* infection (CDI) [1, 2], such as inflammatory bowel disease (IBD) [3–7], serious antibiotics-associated diarrhea in intensive care unit [8], irritable bowel syndrome (IBS) [9, 10], constipation [11], primary sclerosing cholangitis (PSC) [12] and immune checkpoint inhibitor-associated colitis [13]. Methods of FMT delivery are classified into three routes: the upper gut, the mid-gut and the lower gut [14, 15]. The nasogastric tube is a delivering way via the upper gut [16, 17]. The mid-gut route for FMT involves the gastroscopy, nasojejunal tube, percutaneous endoscopic gastro-jejunostomy (PEG-J), oral FMT capsules that dissolve in the mid-gut [15, 18] and mid-gut transendoscopic enteral tubing (TET) [19]. Fecal suspension can be infused into the lower gut through enema, colonoscopy, distal ileum stoma, colostomy and colonic TET [14, 15]. Delivering FMT by colonoscopy is a traditional method, but for patients who need repeated FMTs in a short period of time, they have to endure multiple bowel preparations and colonoscopy. Another way of FMT delivery is by enema, however the bacteria can only reach the rectal and sigmoid colon. In our earlier phase, we once performed FMT through the mid-gut under gastroscopy for IBD patients [20, 21]. However, some patients failed to accept the upper and middle delivering ways due to a psychological burden, the risk of aspiration, and even difficulty breathing [22, 23]. Then, in order to meet the needs of patients with multiple fresh FMTs or whole colon administration of drugs during a period of time, we developed a colonic delivery method by the long-term maintenance of an indwelling, colonoscopically placed transanal enteral tube, which was coined colonic TET [14]. The technique of placing a tube through the anus into cecum for whole colon administration of FMTs or medications is therefore achieved, and it has been successfully used in many hospitals in Asia in recent 2 years [6, 10, 14, 24, 25].

In clinical practice, single FMT could be not be enough, especially in patients with severe CDI [26–28]. The colonic route showed higher efficacy than the mid-gut delivery in CDI [29]. Based on these clinical needs and the limitations of the traditional delivering way via enema, colonic TET as a novel specific drug delivery method has shown its potential advantages. A pilot study by our group demonstrated colonic TET as a safe and convenient procedure for FMT with a 98% of satisfaction in patients in 2016 [14]. However, the potential factors affecting the retention time of TET tube within the colon remains unclear. Therefore, we further recorded data of this prospective study on colonic TET at our center. This study will report the methodology, feasibility, and safety of colonic TET, as well as evaluation of the possible affecting factors on the procedure.

## Methods

### Inclusion and exclusion criteria

This prospective observational study was conducted at the Digestive Disease Center, the Second Affiliated Hospital of Nanjing Medical University, Nanjing, China. Patients who underwent colonic TET for FMT or intracolonic medication administration in our center from October 2014 to November 2018 were included for analysis.

Colonic TET was considered for patients: (1) age ≥ 3-year-old; (2) tolerated colonoscopy; and (3) signed informed consent on TET. Colonic TET was not considered for patients: (1) with severe intestinal stenosis, fistula and risk of perforation during endoscopy; (2) complicated with serious anus lesions which might affect endoscopy; and (3) no suitable location for fixation of the titanium clip onto the intestinal wall, because of severe ulcers or a large number of pseudopolyps. All cases underwent colonic TET were included for the current analysis. This study was conducted under the Declaration of Helsinki, and was approved by the Institutional Ethical Review Board of the Second Affiliated Hospital of Nanjing Medical University. All eligible subjects provided written informed consents.

### Methodology of TET

The concept of colonic TET is to insert a small, soft tube into the deep colon and fix the tube onto the intestinal wall through the anus under endoscopy with endoscopic clips according to our previous report [14]. The endoscopic procedure was shown in the video of colonic TET (Additional file 1). As shown in the supplementary video, the TET tube (2.7 mm outer diameter and 1.8 mm inner diameter, FMT-DT-F-27/1350, FMT medical, Nanjing, China) has three separate loops attached to the tube named “the first site/station” which is at the end of the TET which will be the most proximal site in the colon, “the second site” and “the third site”, which are separated by 10 cm each. Each line-loop on the tube is used to fix the tube onto the intestinal wall with one or two endoscopic clips. There is a guide wire within the tube for TET.


**Additional file 1** Endoscopic procedure of colonic TET was shown in the video 1.


The endoscope is inserted into the target location (including the cecum, ascending colon, transverse colon, descending colon). Then 3–5 ml paraffin oil (medical use level) is injected into endoscopic channel (diameter > 3.2 mm) following with TET tube insertion. The paraffin oil is used to facilitate the removal of the colonoscope over the colonic tube. After the TET tube reaches the target location, the endoscope is taken out under endoscopic view. During this procedure, the assistant stably controls the TET tube until the colonoscope is completely taken out.

**Fig. 1:**
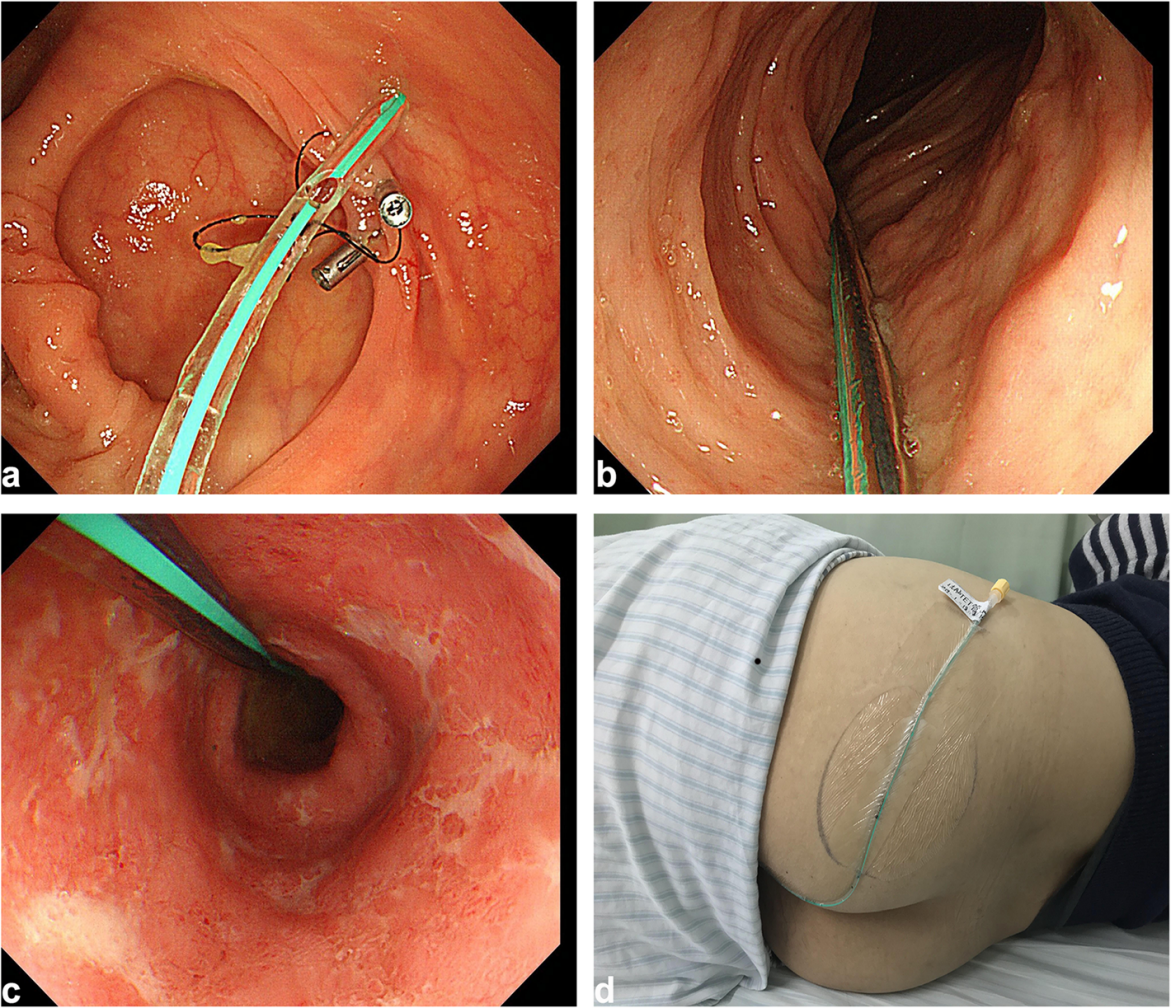
The procedure of colonic TET. **a** Under endoscopic guidance, the distal TET tube was fixed on the colonic wall with two endoscopic clips. **b** The TET tube was within the ascending colon. **c** The TET tube was within the descending colon. **d** The TET tube was fixed onto the skin of the buttocks

As shown in Fig. 1, after the colonoscope is advanced into the target location, one or two disposable endoscopic clips (Large, ROCC-D-26-195-C, ≥ 10 mm, from Nanjing Microtech Co.; Small, HX-610-135 L, 135°, from Olympus) are used to fix the loop of “the 1st site” onto the intestinal wall. Zero to two endoscopic clips can be used for “the 2nd site” and/or “the 3rd site”. We strongly recommend choosing the mucosal fold as the preferred location for clipping for better stability.

During this process, the assistant needs to hold the TET tube for avoiding its removal by the moving endoscope. The biopsy and polypectomy still can be performed after TET if necessary. After the withdrawal of the colonoscope, the extended part of the tube should be separated following the removal of the guide wire. The distal TET tube is finally fixed onto the skin of the hip (preferably on the left hip) with medical tape. The nearest adhesive tape is 5 cm close to the anus. The indwelling tube generally does not affect the defecation and regular life of patients.

### Post-TET management and patient education

The right lateral position is recommended when delivering FMT, medication or tranditional Chinese medication, such as mesalazine solution (60 mL, Salofalk, Losan Pharma GmbH). The liquid or suspension should be injected at a temperature of 37 °C. Patients are required to remain in the right lateral position for at least 30 min after infusion via TET tube, and then allow to choose a comfortable lying position. The angle of head low and foot high is 10°. 5 ml of saline is used for flushing the tube after infusion.

### Clinical evaluation of colonic TET

The treatment purpose, the success rate of the procedure, the fixation location, and the retention time of TET tube, as well as the type and number of endoscopic clips used were recorded. The success rate was defined as the TET tube be successfully fixed to the intestinal wall of patients. The retention time was defined as the time from the implantation of the TET tube to its natural shedding. Adverse events and patients’ satisfaction during and after TET were also investigated. Patient-reported satisfaction on the TET procedure was recorded. The grade of satisfaction was clarified as yes or no [19].

### Statistical analysis

Data were analyzed using SPSS 21.0 (Chicago, IL, USA). When the normality of the distribution of variables was acceptable, independent-samples t-test was used. When the normality of the distribution of variables was not acceptable, the Mann-Whitney U test was used to analyze differences between groups. Comparisons of categorical variables between groups were performed using the Chi-squared test. The retention time of the TET tube was evaluated using univariate and multivariate regression analysis. A value of *p* <  0.05 (two-tailed) was considered to indicate significance.

## Results

### Characteristics of patients

**Fig. 2:**
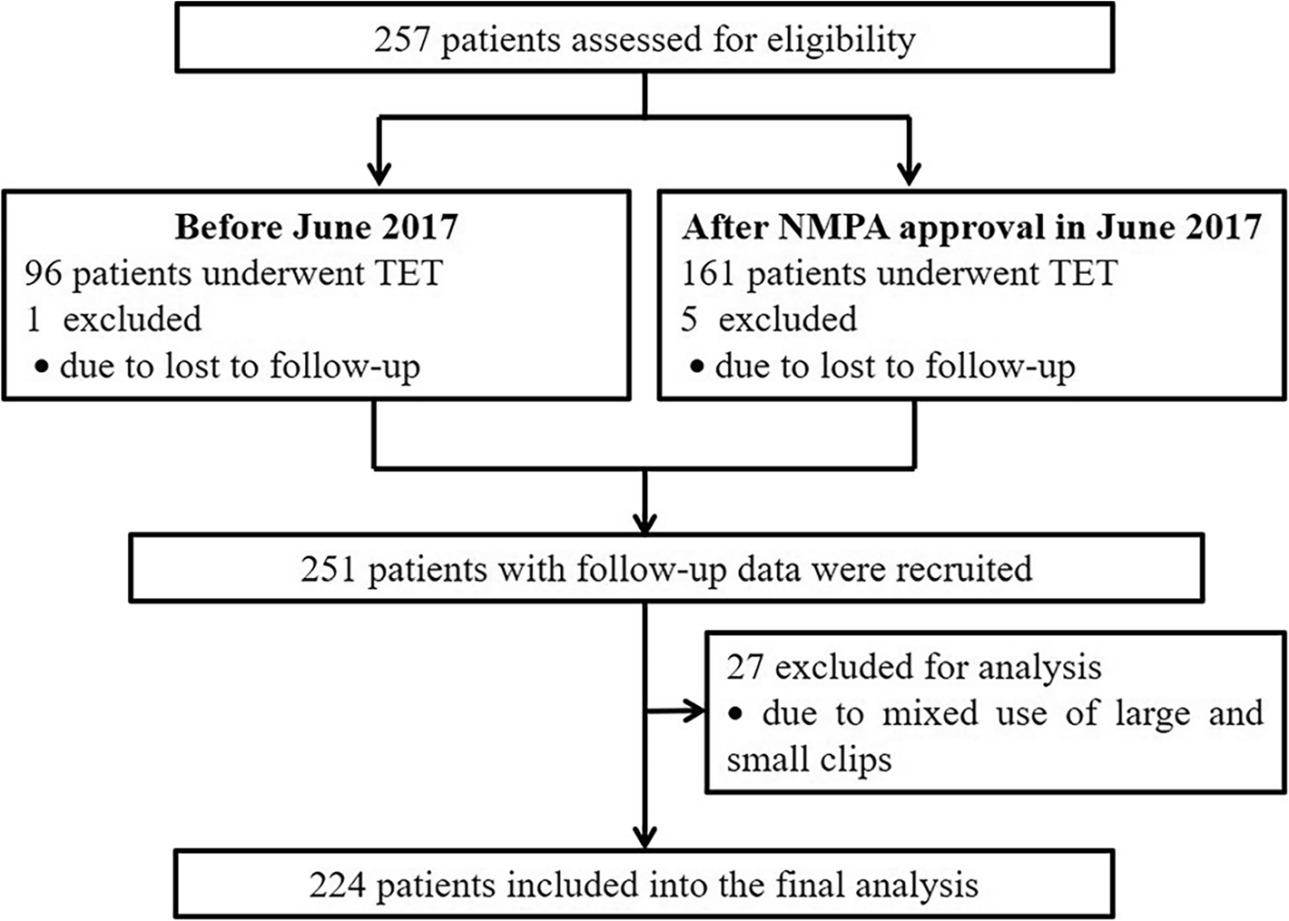
Flow chart of the study. NMPA, National Medical Products Administration

As shown in Fig. 2, a total of 251 patients received colonic TET with complete follow-up data were recruited. 27 patients who received mixed use of large and small endoscopic clips were excluded, and the remaining 224 patients were included in the final analysis of this study. As shown in Table 1, 107 (47.8%) patients used TET for multiple FMTs, 107 (47.8%) for multiple FMTs and intracolonic medication administrations, and 10 (4.5%) for single intracolonic medication administration.

**Table 1:** Characteristics of 224 patients who underwent colonic TET

Items	Results
**Patients, n**	224
**Sex, male, n (%)**	119 (53.1%)
**Age, years, median (IQR)**	40 (28–53)
**Disease type, n (%)**	
UC	118 (52.7%)
Constipation	30 (13.4%)
Others	76 (33.9%)
**Disease duration, years, median (IQR)**	5 (2–9)
**Purpose of TET, n (%)**	
FMT	107 (47.8%)
Colonic medical administration	10 (4.5%)
FMT and medical administration	107 (47.8%)
**Success rate of TET, %**	100%
**Location for fixing distal tube, n (%)**	
Ileocecal	192 (85.6%)
Ascending colon	17 (7.6%)
Transverse colon	5 (2.2%)
Descending colon	6 (2.7%)
Liver curvature	3 (1.3%)
Spleen curvature	1 (0.4%)
**Endoscopic clip type, n (%)**	
Small clip	69, (30.9%)
Large clip	155, (69.1%)
**Endoscopic clip number, median (IQR)**	3 (3–4)
**Retention time of TET tube, median (IQR)**	8.5 (7–11)
**Removal of TET tube, n (%)**	
Spontaneously fell out	158 (70.5%)
Actively pulled out	66 (29.5%)
**Satisfaction, %**	219/224 (97.8%)

*UC* ulcerative colitis, *TET* transendoscopic enteral tubing, *FMT* fecal microbiota transplantation, *IQR* inter quartile range

### Feasibility of colonic TET

The success rate of colon TET was 100% (224/224). The most distal loop site on the TET tube relative to the anus was fixed at the ileocecal junction in 192 patients (85.6%), ascending colon in 17 patients (7.6%), transverse colon in 5 patients (2.2%), and descending colon in 6 (2.7%) patients. During the process of placing the indwelling colonic tube TET, large endoscopic clips were used in 155 patients (69.1%), and small ones in 69 patients (30.9%). The number of endoscopic clips was 3 clips in total (IQR, 3–4). After the treatment was completed, 66 patients (29.5%) were actively pulled out the TET tube. The TET tube spontaneously fell out in 158 patients (70.5%), and the median retention time was 8.5 (IQR 7–11) days. The maximum retention time of the TET tube was up to 28 days.

### Multiple factors analysis on the retention time of TET tube

Among 158 patients with endoscopic clips spontaneously falling out, we analyzed possible influencing factors contributing to the retention time of TET tube. These patients were divided into the short-retention time group (≤ 8.5 days) and the long-retention time group (> 8.5 days). As shown in Table 2, strong associations were observed between TET retention time and the titanium clip type (*p* <  0.001) and the patient age (*p* = 0.026) in the univariate analysis. Multivariate analysis found that only titanium clip type (*p* = 0.001) was an independent factor for affecting the retention time (Table 3). In the subgroup analysis for patients with UC, the univariate and multivariate analysis also demonstrated that the endoscopic clip type (*p* = 0.017) was an independent factor for the retention time of the TET tube.

**Table 2:** Univariate analysis for the retention time of TET tube

Items	Total	Short-retention (≤ 8.5 days)	Long-retention (> 8.5 days)	***P***-value
**Patients (n)**	158	79	79	–
**Sex, male, n**	80	46	34	0.056
**Age, years, m ± SD**	41.6 ± 16.1	38.8 ± 15.5	44.5 ± 16.2	0.026
**Disease type**	158	66	92	0.246
UC	101	47	54	
Others	57	32	25	
**Endoscopic clip type**	158	79	79	< 0.001
Small clip	35	27	8	
Large clip	123	52	71	

In the comparison of groups short-retention and long-retention, disease duration, fixed position and endoscopic clip number were not significantly different (not shown in the table). *SD* Standard deviation, *UC* ulcerative colitis, *IQR* inter quartile range

**Table 3:** Multivariate analysis for the retention time of TET tube

	OR	95% CI	***P***-value
**Sex, male, n**	0.531	0.266–1.061	0.073
**Age, years**	1.015	0.992–1.038	0.202
**Disease type**	1.980	0.962–4.078	0.064
**Endoscopic clip type**	0.208	0.083–0.519	0.001

Further analysis showed that the retention time of TET tube in the large endoscopic clip group was longer than that in the small endoscopic clip group when the number of endoscopic clips used was the same, including three (*p* = 0.002) and four endoscopic clips (*p* = 0.001) total across the three sites. In patients with large endoscopic clips, we found that the number of endoscopic clips used significantly affected their retention time (*p* = 0.013) (Table 4). The retention time of TET tube was significantly prolonged with the increased number of large endoscopic clips. After pairwise comparisons, patients with four endoscopic clips in total had longer retention time than patients with two endoscopic clips in total (adjusted *p* = 0.047); and also longer than patients with three endoscopic clips (adjusted *p* = 0.030). In patients with small endoscopic clips, the retention time of the TET tube did not show a significant change with an increased number of endoscopic clips (*p* = 0.498).

**Table 4:** Correlation between the titanium clip number and TET retention time

	Endoscopic clip number	Frequency	TET retention time	***P***-value
**Small clip**	3	7	6 (4–7)	
	4	9	7 (6.5–7.5)	
	5	10	7.5 (5–10.5)	
	6	9	7 (5.5–9.5)	0.498
**Large clip**	2	14	7.5 (6–11)	
	3	72	9 (7–11)	
	4	35	11 (8–12)	0.013

### Satisfaction and safety of the colonic TET

The patients’ satisfaction rate for colonic TET was 97.8% (219/224). No severe adverse event was observed during and after colonic TET. Among all patients with colonic TET, total 8.0% (18/224) of patients complained about adverse events and all of them were mild.

No obstruction occurred during and after infusion via the TET tube. One case reported mild abdominal pain, but the abdominal pain relieved after the infusion. 3% of cases (2/66) reported abdominal discomfort during the procedure of removing the TET tube.

## Discussion

Colonic TET, as a new approach for colon-targeted drug delivery, has shown its promising potential in treating intestinal diseases, such as UC and CDI. The present prospective study, as the largest number, reported 97.8% of satisfaction with the colonic TET. We found that the retention time of the colonic TET tube was significantly correlated with the endoscopic clip type. When the number of endoscopic clips used was three or four, the retention time of the TET tube using large endoscopic clips was significantly longer than that of small endoscopic clips. In patients who used large endoscopic clips, our results showed that as the number of endoscopic clips used increased, the retention time of the TET tube became longer. However, when the number of endoscopic clips was over five, the retention time was no longer extended consistently. This indicates that the number of endoscopic clips used during TET should not exceed five, because the increased number may not bring more benefits to patients, but result in an increase in medical cost.

The retention time of the TET tube is related to the doctor’s clinical decision on the patient’s condition. According to the retention time of the TET tube, it is recommended to use 2–3 large endoscopic clips in total to fix TET tube in patients who need multiple FMTs or colonic administration of drugs such as mesalamine in a short period of time. For patients with UC who are severe or complicated with extensive colonic ulcers, multiple FMTs or a long-term intracolonic administration of drugs is required. At this time, the TET tube should be retained for as long as possible, and it should be fixed with 3–4 large endoscopic clips in total. For patients who only need 1 to 2 FMTs and do not need a long-term intracolonic administration of drugs, 1–2 large endoscopic clips in total should be selected to fix the TET tube. However, further studies are necessary to answer the limitation and benefits using one endoscopic clip for the fixation of TET tube.

Colonic TET can be used for the whole colon administration of drugs. Traditionally, the drugs in liquid or suspension can only be delivered into the rectum about 20 cm through an enema. Colonic TET is a new option for patients with intestinal diseases, especially for IBD, who need the treatment of drugs with mesalamine, steroids or traditional herbs. There was no tube obstruction when the tube was flushed actively after washed microbiota transplantation or administration of the drugs in the present study [30]. However, the tube obstruction was reported in another pilot study during delivering manual prepared fecal suspension [24]. The fixation locations of the TET tube should be decided by endoscopists according to the patients’ intestinal condition. The TET tube was placed at the cecum in most patients. The TET tubes were fixed at the transverse colon in 5 patients, because of the difficulty or risk for the endoscope arriving at cecum. The TET tube was fixed at the descending colon in 6 patients only after common cleansing enema. Therefore, the sites of the TET tube can be selectively fixed at the targeted intestinal section to meet different treatment requirements.

This evaluation indicated that the colonic TET should be a simple technique with a high degree of success for patients. However, the colonic TET is not recommended in the cases with obvious narrow stenosis, deep ulceration or obvious edema in the intestinal wall, in case of perforation. The tips for having a successful procedure at least include: the clip should cover thick wall tissue (e.g. plica) before closing; the two fixation sites/stations cannot be too tight in order to avoid traction; once the clipping is done, withdraw the colonoscope while gently rotating it to avoid the coiling of the tube around the colonoscope or the tube expulsion. When two to four large clips are used, the maintaining time becomes proportionately longer with the more clips used. Therefore, it should be useful for maintaining a TET tube for a median of over 7 days if using 2–4 large clips.

There are several limitations. This article mainly reported the methodology and possible reasons affecting the colonic TET procedure. The feasibility of using one endoscopic clip was not evaluated in the present study. The use of TET in children under 7 years old should be further carefully evaluated. Patients’ disease severity and treatment efficacy were not included for analysis. It lacks a comparison between colonic TET and other colon-specific drug delivery methods. The cost-effectiveness analysis of colonic TET should be further conducted. The relationship between the type and number of endoscopic clips and the retention time should be evaluated in a larger sample size. Randomized controlled trials for further evaluation of this novel technique is necessary.

## Conclusions

In conclusion, colonic TET as a novel colon specific drug delivery method is a feasible, practical, and safe technique with a high degree of patients’ satisfaction for multiple FMTs or frequent colonic medication administration. Generally, two to four large endoscopic clips are recommended to ensure the fixation of the TET tube onto the colonic wall and maintain it for over 7 days. However, using endoscopic clips over 4 would not contribute to prolonging the maintaining time of the TET tube. This study provides a rationale for colonic TET to be developed as a novel approach for multiple FMTs or whole colon administration of drugs for the treatment of diseases.

## Data Availability

The datasets generated and analyzed during the current study are available from the corresponding author on reasonable request.

## Abbreviations

FMT: Fecal microbiota transplantation
IBD: Inflammatory bowel diseases
UC: Ulcerative colitis
TET: Transendoscopic enteral tubing

## References

[1] Mullish BH, Quraishi MN, Segal JP, McCune VL, Baxter M, Marsden GL, Moore DJ, Colville A, Bhala N, Iqbal TH (2018). The use of faecal microbiota transplant as treatment for recurrent or refractory Clostridium difficile infection and other potential indications: joint British Society of Gastroenterology (BSG) and Healthcare Infection Society (HIS) guidelines. Gut.

[2] McDonald LC, Gerding DN, Johnson S, Bakken JS, Carroll KC, Coffin SE, Dubberke ER, Garey KW, Gould CV, Kelly C (2018). Clinical practice guidelines for Clostridium difficile infection in adults and children: 2017 update by the Infectious Diseases Society of America (IDSA) and Society for Healthcare Epidemiology of America (SHEA). Clin Infect Dis.

[3] Paramsothy S, Kamm MA, Kaakoush NO, Walsh AJ, van den Bogaerde J, Samuel D, Leong RWL, Connor S, Ng W, Paramsothy R (2017). Multidonor intensive faecal microbiota transplantation for active ulcerative colitis: a randomised placebo-controlled trial. Lancet.

[4] Costello SP, Hughes PA, Waters O, Bryant RV, Vincent AD, Blatchford P, Katsikeros R, Makanyanga J, Campaniello MA, Mavrangelos C (2019). Effect of fecal microbiota transplantation on 8-week remission in patients with ulcerative colitis: a randomized clinical trial. JAMA.

[5] Wang H, Cui B, Li Q, Ding X, Li P, Zhang T, Yang X, Ji G, Zhang F (2018). The safety of fecal microbiota transplantation for Crohn's disease: findings from a Long-term study. Adv Ther.

[6] Ding X, Li Q, Li P, Zhang T, Cui B, Ji G, Lu X, Zhang F (2019). Long-term safety and efficacy of fecal microbiota transplant in active ulcerative colitis. Drug Saf.

[7] Li P, Zhang T, Xiao Y, Tian L, Cui B, Ji G, Liu YY, Zhang F (2019). Timing for the second fecal microbiota transplantation to maintain the long-term benefit from the first treatment for Crohn's disease. Appl Microbiol Biotechnol.

[8] Dai M, Liu Y, Chen W, Buch H, Shan Y, Chang L, Bai Y, Shen C, Zhang X, Huo Y (2019). Rescue fecal microbiota transplantation for antibiotic-associated diarrhea in critically ill patients. Critical care (London, England).

[9] Ianiro G, Eusebi LH, Black CJ, Gasbarrini A, Cammarota G, Ford AC (2019). Systematic review with meta-analysis: efficacy of faecal microbiota transplantation for the treatment of irritable bowel syndrome. Aliment Pharmacol Ther.

[10] Huang HL, Chen HT, Luo QL, Xu HM, He J, Li YQ, Zhou YL, Yao F, Nie YQ, Zhou YJ (2019). Relief of irritable bowel syndrome by fecal microbiota transplantation is associated with changes in diversity and composition of the gut microbiota. J Dig Dis.

[11] Tian H, Ding C, Gong J, Ge X, McFarland LV, Gu L, Wei Y, Chen Q, Zhu W, Li J (2016). Treatment of slow transit constipation with fecal microbiota transplantation: a pilot study. J Clin Gastroenterol.

[12] Allegretti JR, Kassam Z, Carrellas M, Mullish BH, Marchesi JR, Pechlivanis A, Smith M, Gerardin Y, Timberlake S, Pratt DS, et al. Fecal microbiota transplantation in patients with primary Sclerosing cholangitis: a pilot clinical trial. Am J Gastroenterol. 2019;114(7):1071–9. 10.14309/ajg.0000000000000115 30730351

[13] Wang Y, Wiesnoski DH, Helmink BA, Gopalakrishnan V, Choi K, DuPont HL, Jiang ZD, Abu-Sbeih H, Sanchez CA, Chang CC (2018). Fecal microbiota transplantation for refractory immune checkpoint inhibitor-associated colitis. Nat Med.

[14] Peng Z, Xiang J, He Z, Zhang T, Xu L, Cui B, Li P, Huang G, Ji G, Nie Y (2016). Colonic transendoscopic enteral tubing: a novel way of transplanting fecal microbiota. Endoscopy international open.

[15] Zhang F, Cui B, He X, Nie Y, Wu K, Fan D. Group FM-sS: microbiota transplantation: concept, methodology and strategy for its modernization. Protein Cell. 2018;9(5):462–73. 10.1007/s13238-018-0541-8 PMC5960466 29691757

[16] Postigo R, Kim JH (2012). Colonoscopic versus nasogastric fecal transplantation for the treatment of Clostridium difficile infection: a review and pooled analysis. Infection.

[17] Youngster I, Sauk J, Pindar C, Wilson RG, Kaplan JL, Smith MB, Alm EJ, Gevers D, Russell GH, Hohmann EL (2014). Fecal microbiota transplant for relapsing Clostridium difficile infection using a frozen inoculum from unrelated donors: a randomized, open-label, controlled pilot study. Clin Infect Dis.

[18] Ni X, Fan S, Zhang Y, Wang Z, Ding L, Li Y, Li J (2016). Coordinated hospital-home fecal microbiota transplantation via percutaneous endoscopic Cecostomy for recurrent steroid-dependent ulcerative colitis. Gut Liver.

[19] Long C, Yu Y, Cui B, Jagessar SAR, Zhang J, Ji G, Huang G, Zhang F (2018). A novel quick transendoscopic enteral tubing in mid-gut: technique and training with video. BMC Gastroenterol.

[20] Cui B, Feng Q, Wang H, Wang M, Peng Z, Li P, Huang G, Liu Z, Wu P, Fan Z (2015). Fecal microbiota transplantation through mid-gut for refractory Crohn's disease: safety, feasibility, and efficacy trial results. J Gastroenterol Hepatol.

[21] Cui B, Li P, Xu L, Zhao Y, Wang H, Peng Z, Xu H, Xiang J, He Z, Zhang T (2015). Step-up fecal microbiota transplantation strategy: a pilot study for steroid-dependent ulcerative colitis. J Transl Med.

[22] Anderson JL, Edney RJ, Whelan K (2012). Systematic review: faecal microbiota transplantation in the management of inflammatory bowel disease. Aliment Pharmacol Ther.

[23] Colman RJ, Rubin DT (2014). Fecal microbiota transplantation as therapy for inflammatory bowel disease: a systematic review and meta-analysis. J Crohns Colitis.

[24] Wang JW, Wang YK, Zhang F, Su YC, Wang JY, Wu DC, Hsu WH (2019). Initial experience of fecal microbiota transplantation in gastrointestinal disease: a case series. Kaohsiung J Med Sci.

[25] Xie WR, Yang XY, Xia HH, Wu LH, He XX (2019). Hair regrowth following fecal microbiota transplantation in an elderly patient with alopecia areata: a case report and review of the literature. World J Clin Cases.

[26] Ianiro G, Masucci L, Quaranta G, Simonelli C, Lopetuso LR, Sanguinetti M, Gasbarrini A, Cammarota G (2018). Randomised clinical trial: faecal microbiota transplantation by colonoscopy plus vancomycin for the treatment of severe refractory Clostridium difficile infection-single versus multiple infusions. Aliment Pharmacol Ther.

[27] Ianiro G, Valerio L, Masucci L, Pecere S, Bibbo S, Quaranta G, Posteraro B, Curro D, Sanguinetti M, Gasbarrini A (2017). Predictors of failure after single faecal microbiota transplantation in patients with recurrent *Clostridium difficile* infection: results from a 3-year, single-centre cohort study. Clin Microbiol Infect.

[28] Fischer M, Sipe BW, Rogers NA, Cook GK, Robb BW, Vuppalanchi R, Rex DK (2015). Faecal microbiota transplantation plus selected use of vancomycin for severe-complicated Clostridium difficile infection: description of a protocol with high success rate. Aliment Pharmacol Ther.

[29] Ianiro G, Maida M, Burisch J, Simonelli C, Hold G, Ventimiglia M, Gasbarrini A, Cammarota G (2018). Efficacy of different faecal microbiota transplantation protocols for Clostridium difficile infection: a systematic review and meta-analysis. United European Gastroenterol J.

[30] Zhang T, Lu G, Zhao Z, Liu Y, Shen Q, Li P, Chen Y, Yin H, Wang H, Marcella C, et al. Washed microbiota transplantation vs. manual fecal microbiota transplantation: clinical findings, animal studies and in vitro screening. Protein Cell. 2020;11(4):251–66. 10.1007/s13238-019-00684-8 PMC7093410 31919742

